# BactoRamanBioNet: A Multimodal Neural Network for Bacterial Species Identification Using Raman Spectroscopy and Biological Knowledge

**DOI:** 10.3390/s26061828

**Published:** 2026-03-13

**Authors:** Yaoxue Xu, Junzhuo Song, Zhen Zhang, Lin Feng, Yalan Yang, Yunsen Liang, Yan Guo

**Affiliations:** 1 College of Information Engineering, Sichuan Agricultural University, Ya’an 625014, China; xuyaoxue@stu.sicau.edu.cn (Y.X.); zhangzhen2@stu.sicau.edu.cn (Z.Z.); 202308664@stu.sicau.edu.cn (Y.Y.); 202308549@stu.sicau.edu.cn (Y.L.); 2 College of Water Resources and Hydropower, Sichuan Agricultural University, Ya’an 625014, China; 202307883@stu.sicau.edu.cn; 3 College of Life Science, Sichuan Agricultural University, Ya’an 625014, China; fenglin@stu.sicau.edu.cn

**Keywords:** multimodal data, deep learning, bacterial identification, Raman spectroscopy, Transformer architecture

## Abstract

Accurate and rapid identification of bacterial species is essential for public health, clinical diagnostics, and environmental monitoring. Although Raman spectroscopy offers a powerful, non-invasive alternative, reliance solely on spectral data often fails to distinguish species with highly similar signatures, particularly when the discriminating features are subtle. This difficulty is frequently compounded by a lack of integrated biological prior knowledge, which can hinder model performance. To address these challenges, we introduce BactoRamanBioNet, a novel multimodal neural network architecture. Our model employs a synergistic approach that utilizes a ResNet-Transformer architecture to capture complex spectral patterns and a CLIP text encoder to incorporate descriptive biological information, thereby enabling highly accurate multimodal classification of bacterial species. Empirical results demonstrate that BactoRamanBioNet achieves a classification accuracy of 98.2% and an F1-score of 98.0%. This performance surpasses the current state-of-the-art deep learning model, ResNet-1D, by 2.4% in accuracy and 2.0% in F1-score. Moreover, our model outperforms traditional classifiers, such as Support Vector Machine (SVM) and Random Forest (RF), by 9.8% and 7.9% in accuracy, respectively, while also exhibiting significant improvements in precision and recall. By establishing a new benchmark in performance and robustness, BactoRamanBioNet offers a powerful and reliable framework for automated microbiological analysis, paving the way for next-generation diagnostic systems.

## 1. Introduction

Accurate identification of bacterial species is essential in public health, clinical diagnostics, and environmental monitoring. Traditional methods for bacterial identification typically depend on morphological observation, biochemical tests, and molecular biology techniques, including the polymerase chain reaction (PCR) and gene sequencing. However, these approaches are often time-consuming, expensive, and require highly skilled personnel. In recent years, Raman spectroscopy, due to its ability to directly detect molecular vibrational modes, serves as a powerful biological sensor for bacterial analysis. The technique allows for the non-invasive identification and classification of bacteria by capturing the vibrational characteristics of bacterial cells, such as the composition of proteins, lipids, and nucleic acids. This characteristic makes Raman spectroscopy a highly effective tool for real-time detection of bacterial species, especially when compared to traditional methods, which are often time-consuming, require culture media, and are dependent on laboratory conditions. By providing detailed spectral information without the need for chemical reagents or complex sample preparation, Raman spectroscopy offers a rapid and cost-effective alternative for microbial identification, functioning as an on-site biological sensor. This makes it especially suitable for applications in clinical diagnostics, environmental monitoring, and food safety, where rapid, accurate, and non-invasive detection of bacterial contamination is crucial. In the context of microbial analysis, Raman spectroscopy as a biological sensor offers significant advantages for monitoring pathogen presence in various environments and early detection of infections, while also providing insights into the biological state of bacteria—something traditional methods struggle to capture. Raman spectroscopy has emerged as an efficient analytical technique for microbial identification, owing to its non-invasive nature, rapid execution, and high sensitivity. By employing Raman spectroscopy, the molecular vibrational characteristics of bacteria can be precisely captured, facilitating species differentiation and classification [1,2].

In the realm of Raman spectroscopy data analysis, machine learning (ML) has been extensively utilized for the classification of Raman spectral data. Raman spectroscopy is a powerful analytical technique based on the inelastic scattering of light, where the interaction between photons and molecular vibrations provides characteristic spectral information about the chemical composition and molecular structure of samples. The fundamental principle of Raman spectral analysis lies in the fact that each molecular bond possesses unique vibrational modes, producing distinct spectral peaks at specific wavenumbers. The intensity of these spectral peaks is directly related to the concentration of corresponding chemical components and their molecular environment, thereby reflecting the biological characteristics of the studied samples. For instance, spectral peaks associated with proteins, lipids, nucleic acids, and carbohydrates exhibit varying intensities depending on biochemical composition, enabling the differentiation between healthy and diseased tissues or distinct cell types. In the medical field, Raman spectroscopy has been widely applied for disease diagnosis, particularly in cancer detection, where analysis of Raman spectra from tissue samples can distinguish tumor cells from healthy cells [3]. Moreover, Raman spectroscopy has found applications in microbiology, enabling pathogen identification and rapid diagnosis through analysis of bacterial and viral Raman spectra [4]. In Alzheimer’s disease research, the combination of explainable machine learning with graphene-assisted Raman spectroscopy enables rapid biomarker screening. This study demonstrates how integrating machine learning algorithms with the unique properties of graphene-assisted Raman spectroscopy significantly improves the efficiency and accuracy of biomarker screening [5,6] During the global COVID-19 pandemic, surface-enhanced Raman scattering (SERS) combined with machine learning was employed to study the SARS-CoV-2 receptor-binding domain. The integration of SERS with machine learning provides a powerful tool for detecting and analyzing the SARS-CoV-2 receptor-binding domain. This research not only showcases the practical application of machine learning-assisted Raman spectroscopy in virology but also highlights its potential in addressing public health emergencies [7]. Furthermore, machine learning-enhanced hyperspectral Raman imaging plays a significant role in label-free molecular mapping of Alzheimer’s disease brains. Hyperspectral Raman imaging combined with machine learning can generate comprehensive label-free molecular maps of Alzheimer’s disease brains, facilitating better understanding of the complex molecular changes associated with Alzheimer’s disease and further emphasizing the versatility of machine learning-assisted Raman spectroscopy in neuroscience research [8]. In environmental monitoring, Raman spectroscopy is employed for detecting pollutants in water and air, and assessing their concentrations [9]. Similarly, in the food industry, Raman spectroscopy is used for food quality control and authentication, ensuring product authenticity and safety [10,11] Traditional machine learning approaches, such as support vector machines (SVMs), have demonstrated considerable efficacy in Raman spectral classification, particularly when combined with suitable kernel functions. SVM operates by maximizing the margin between categories to identify the optimal boundary, rendering it adept at managing high-dimensional data. However, SVM is contingent upon manual feature selection and may face challenges when addressing highly complex or non-linear spectral data [12,13]. In contrast, Random Forest (RF) is another prevalent method for classifying Raman spectral data. RF constructs multiple decision trees and amalgamates them, thereby enhancing the model’s robustness and accuracy. Unlike SVM, RF does not depend on manual feature selection and is more adept at managing complex data. Its capability to accommodate a large number of input variables, coupled with a strong resistance to overfitting, has enabled Random Forest to excel in numerous spectral analysis applications [14,15,16]. However, these machine learning methods often necessitate specialized data preprocessing and domain expertise to achieve optimal performance [17].

In contrast, deep learning models can automatically capture the non-linear complexities within datasets and identify subtle patterns that traditional methods may overlook, eliminating the need for extensive data preprocessing. This capability positions deep learning as an ideal candidate for fully leveraging the potential of Raman spectroscopy [18]. The integration of single-cell Raman spectroscopy with deep learning presents a promising approach for rapid, cultivation-free bacterial identification. By training models on the Raman spectra of pathogenic bacteria and evaluating bacterial types within the training set, deep learning can discern subtle differences in Raman spectral fingerprints at both the species and strain levels. PSE-LR (Position-Sensitive Embedding with Logistic Regression), SpecTr (Spectral Transformer), and RamanNet are three specialized architectures that have been developed to address the unique challenges of spectral data analysis [19,20,21]. PSE-LR (logistic regression with peak-sensitive elastic-net regularization) is an algorithm specifically tailored for high-dimensional spectral classification. By generating peak-sensitive feature importance maps, PSE-LR simultaneously achieves both high classification accuracy and interpretability. SpecTr, a Transformer-based model specifically designed for spectral data, leverages the power of attention mechanisms to handle complex spectral dependencies, allowing it to process and learn from spectral sequences more effectively. RamanNet, on the other hand, is a deep learning architecture tailored for Raman spectral analysis, designed to automatically extract high-level features and patterns from Raman spectra, enhancing the accuracy of bacterial species identification. Convolutional neural networks (CNNs) have been extensively utilized for single-modal spectral recognition [22,23]. Existing studies predominantly utilize traditional single-modal spectral analysis methods, which frequently neglect noise and complexity inherent in the spectral data. This oversight restricts the accuracy and stability of classification outcomes. To enhance classification accuracy, some researchers have sought to improve spectral data quality through signal processing techniques, including denoising, smoothing, and feature extraction. However, single-modal spectral information continues to be inadequate in addressing the challenges presented by the complexity of bacterial species and the variability of experimental environments [24,25].

Research increasingly emphasizes multimodal approaches that integrate biological knowledge with Raman spectral data [26,27]. Some investigators have employed multimodal learning techniques that merge Raman spectral data with other imaging modalities to enhance the accuracy and robustness of bacterial classification [28,29]. However, the majority of datasets lack multiple modalities, and the acquisition of such data is often prohibitively expensive, thereby constraining the applicability of multimodal methods. Consequently, a significant research challenge lies in effectively extracting information from Raman spectral data and seamlessly integrating it with the existing biological knowledge contained within the data to develop a model characterized by high classification accuracy and robustness.

To address these challenges, this study introduces a novel multimodal neural network model, BactoRamanBioNet, which innovatively combines Raman spectroscopy and biological knowledge for automated bacterial species identification. Unlike traditional unimodal models, BactoRamanBioNet overcomes the limitations of single-source data by integrating Raman spectral data with bacterial feature peak knowledge. Specifically, the Raman spectral data provide molecular-level spectral features of bacteria, while the biological knowledge of bacterial feature peaks offers additional domain understanding. This fusion not only enhances the model’s interpretability of spectral data but also improves its generalization ability to unseen data.

The innovation of BactoRamanBioNet lies in its adoption of a multimodal fusion framework, which effectively combines spectral data with biological prior knowledge. Traditional CNN or CNN-Transformer hybrid architectures typically focus on extracting features from single-modal data, often neglecting the integration of external domain knowledge to enhance feature representation. In contrast, our approach incorporates biological domain knowledge, significantly improving the model’s interpretability. This allows the model to not only learn discriminative features from the spectral data but also to understand the biological significance behind these features. This methodological innovation enables BactoRamanBioNet to achieve superior classification accuracy and reduced error rates in bacterial species identification tasks, especially when confronted with diverse and complex spectral data.

Compared to unimodal models, BactoRamanBioNet demonstrates significant performance improvements. Experimental results show that BactoRamanBioNet achieves higher accuracy and lower error rates in bacterial species identification tasks under the same dataset and experimental settings. This performance enhancement is not only a result of optimization but also reflects the innovative application of multimodal data fusion and biological knowledge-guided learning. Therefore, BactoRamanBioNet represents a significant advancement in bacterial species identification and provides new insights and methodologies for the application of deep learning in the biological field.

## 2. Related Works

### 2.1. Traditional Machine Learning Methods

Traditional machine learning methods have been widely applied in bacterial species identification, particularly in the analysis of Raman spectroscopy data. The core idea of these methods is to extract handcrafted features from raw spectral data and use classification algorithms for modeling. The main advantages of traditional methods lie in their strong interpretability and relatively low computational requirements; however, they are highly dependent on the quality of the data and the feature extraction process [30].

Common traditional machine learning methods include Support Vector Machines (SVM), Decision Trees, and Random Forests (RF), all of which have been extensively used in Raman spectral classification tasks. SVM, due to its characteristic of finding the maximum margin hyperplane in high-dimensional spaces, is effective in handling the complexity and high-dimensional features of Raman spectral data [31]. Li et al. applied the SVM model to classify bacteria using Raman spectra and achieved good results, demonstrating the effectiveness of SVM in bacterial recognition [32]. Particularly in cases with limited sample sizes, SVM can still maintain high classification accuracy, making it a common and effective classification method [23].

In addition, Random Forest (RF), as an ensemble learning method, improves classification robustness and accuracy by constructing multiple decision trees and aggregating their results. RF models can effectively avoid overfitting and handle high-dimensional complex data by randomly selecting feature and data subsets. Zhang et al. used the RF algorithm to classify bacterial Raman spectra, achieving better classification accuracy than traditional single decision trees [33]. Since RF does not rely on a single feature selection and is capable of handling non-linear data, it is frequently applied to Raman spectral data. However, it still depends on manually designed features, limiting its performance with complex data [34].

Although these traditional machine learning methods have achieved considerable results in Raman spectral classification, they typically rely on manual feature extraction and selection, which requires significant domain expertise. Even with advanced signal processing techniques such as denoising and smoothing, these methods still struggle to capture the full spectrum of potential complex patterns from raw spectral data. While traditional machine learning methods perform well with relatively simple and standardized spectral data, they exhibit limitations when dealing with high data complexity and diverse samples. Furthermore, traditional machine learning methods are unable to automatically perform deep feature learning, making it difficult for them to fully explore the deep patterns within high-dimensional and non-linear spectral data [20].

### 2.2. Deep Learning Methods

With the rapid development of deep learning technology, an increasing number of researchers have started employing deep neural networks (DNNs) and convolutional neural networks (CNNs) for automatic feature learning and classification of Raman spectral data. The main advantage of deep learning methods is their ability to automatically extract complex features from data, eliminating the need for manual feature extraction, thereby improving the accuracy and robustness of bacterial classification [35].

Convolutional neural networks (CNNs), in particular, have shown significant advantages in processing image data, which has led to widespread attention to their application in Raman spectral analysis. CNNs can capture local features and effectively identify important patterns within the spectra, making them particularly suitable for classification tasks involving Raman spectra. Especially when Raman spectral data exhibits high dimensionality and non-linearity, deep learning methods can uncover subtle patterns that traditional methods cannot detect [36]. For example, Wang et al. employed deep convolutional neural networks (CNNs) for bacterial Raman spectral data classification, achieving higher classification accuracy compared to traditional methods, especially when the number of bacterial samples was limited. CNNs were better at extracting useful features from complex data [37].

Additionally, deep neural networks (DNNs) have been widely used for Raman spectral data classification. Their multi-layer network structure allows them to gradually extract features from the data, making them especially suitable for high-dimensional data processing. For instance, Liu et al. proposed a DNN-based bacterial Raman spectral classification method, which outperformed traditional machine learning methods, particularly with complex Raman spectral datasets [38].

While deep learning methods have shown remarkable performance improvements in bacterial identification, they still face certain challenges. First, deep learning methods typically require large amounts of labeled data for training, and obtaining labeled data for bacterial species identification is an expensive and time-consuming process. Zhang et al. explored the dependence of deep learning on labeled data and proposed a semi-supervised learning approach to reduce the need for labeled data, achieving promising results in their experiments [39]. Moreover, deep learning models require high computational resources, particularly when training complex networks, which makes them less accessible for use in resource-limited laboratory settings [40]. Additionally, deep learning models are sensitive to noise in the spectral data, which may lead to a decline in classification accuracy. To address this issue, some researchers have employed data augmentation and denoising methods to improve model stability [41,42].

Despite these challenges, the advantages of deep learning methods remain undeniable. By continuously optimizing model structures and employing advanced techniques such as transfer learning and reinforcement learning, deep learning methods are expected to further improve performance in future bacterial classification studies and expand to more complex biological data analysis tasks.

### 2.3. Multimodal Methods

To further improve bacterial classification accuracy and stability, researchers have increasingly explored multimodal learning methods in recent years. Multimodal learning integrates data from different modalities (e.g., Raman spectral data, genomic data, imaging data, etc.), leveraging the strengths of each modality to provide more comprehensive and accurate information. By fusing different data sources, the model can recognize more enriched features, greatly enhancing its ability to identify bacterial species [43]. Compared to single-modality data, multimodal methods can handle more complex biological information, offering stronger robustness and generalization ability.

In the application of Raman spectroscopy, more and more researchers have adopted multimodal approaches to combine data from various sources. For instance, Zhou et al. proposed an innovative multimodal deep learning model that combines Raman spectral data with bacterial gene expression data, significantly improving classification accuracy and robustness [24]. Through multimodal learning, the model can acquire rich feature information from multiple perspectives and data sources, thereby better understanding the molecular characteristics of bacteria. This approach not only improves classification accuracy but also reduces noise and bias that may exist in single-modality data.

Additionally, some studies have combined Raman spectral data with imaging data, using deep neural networks for integrated analysis. For example, Liu et al. proposed a deep learning model that integrates Raman spectroscopy with microscopy imaging data, improving bacterial classification accuracy and efficiency [44]. While multimodal methods have demonstrated great potential, they still face challenges in data integration and processing. Preprocessing, alignment, and fusion of different modality data remain difficult tasks in multimodal learning. Moreover, acquiring and processing multimodal data is typically more complex and expensive than working with single-modality data. Therefore, how to effectively extract multimodal information, reduce data acquisition costs, and improve Raman spectral recognition accuracy remains an important issue in current research.

## 3. Methods

**Figure 1:**
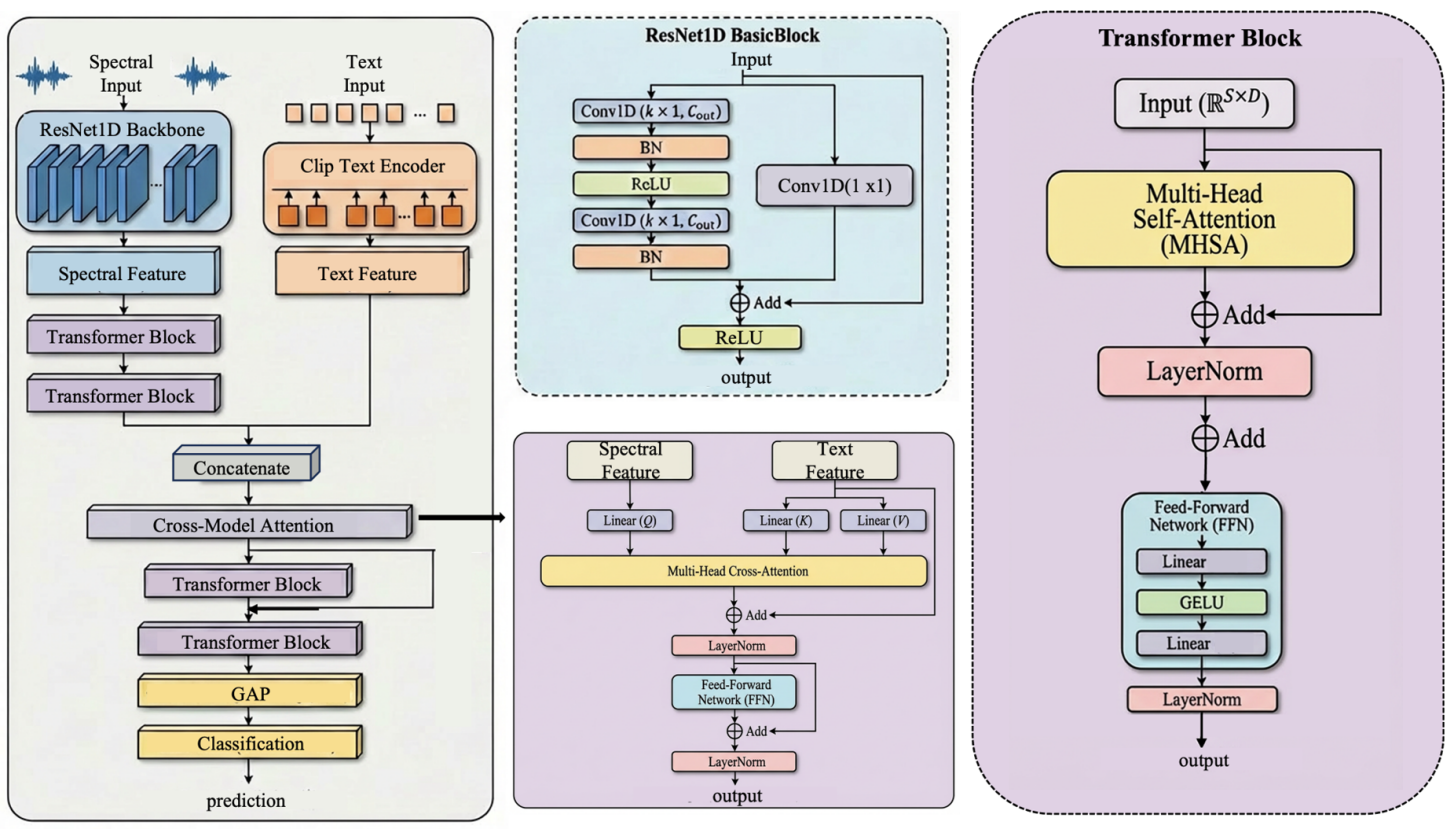
The overall architecture of BactoRamanBioNet. The model is structured into three primary modules: the spectral feature encoding module, the Transformer-based feature modeling module, and the multimodal feature fusion and prediction module.

The BactoRamanBioNet architecture represents a multimodal deep learning framework aimed at achieving robust and accurate classification of bacterial species. This model integrates spectral and textual data, capitalizing on their complementary strengths to improve predictive performance. The architecture is systematically structured into three primary modules that operate sequentially: a spectral feature encoding module, a Transformer-based feature modeling module, and a multimodal feature fusion and prediction module, as conceptually depicted in Figure 1.

The initial stage of our model processes the input Raman spectral data, denoted as a sequence

(1) $$X=\{x_{1},x_{2},\ldots ,x_{N}\}$$

where *N* is the number of spectral points. To extract high-level, discriminative feature representations from this data, we employ a Residual Network (ResNet). The choice of ResNet is motivated by its demonstrated efficacy in capturing complex, hierarchical patterns while mitigating the gradient vanishing problem common in deep neural networks. A fundamental component of the ResNet is the residual block, which is mathematically formulated as follows:

(2) $$x_{l+1}=\sigma \left(F\left(x_{l},\left\{W_{l}\right\}\right)+x_{l}\right)$$

Here, $x_{l}$ and $x_{l+1}$ are the input and output feature maps of the *l*-th residual block, respectively. F represents the residual mapping learned by the block’s convolutional layers with weights $\left\{W_{l}\right\}$, and σ denotes a non-linear activation function, such as ReLU. This residual learning paradigm facilitates the training of deeper networks. The entire encoding process can be abstracted as:

(3) $$Z=\mathrm{ResNet}(X)\in R^{L\times D}$$

where *Z* represents the resulting sequence of deep spectral features, with *L* as the feature map’s sequence length and *D* as the feature dimension.

Following the initial feature extraction, a Transformer-based feature modeling module is introduced to effectively model the global dependencies and contextual relationships among the extracted spectral features. This module leverages the self-attention mechanism to weigh the significance of different features within the sequence *Z*. The core of this mechanism is the scaled dot-product attention, which is computed as follows:

(4) $$\mathrm{Attention}(Q,K,V)=\mathrm{softmax}\left(\frac{QK^{T}}{\sqrt{d_{k}}}\right)V$$

In this formulation, the query (*Q*), key (*K*), and value (*V*) matrices are linear projections of the input feature sequence: $Q=ZW^{Q}$, $K=ZW^{K}$, and $V=ZW^{V}$, where $W^{Q},W^{K},W^{V}$ are learnable weight matrices and $d_{k}$ is the dimension of the key vectors used for scaling. By stacking multiple self-attention heads and feed-forward networks, the Transformer encoder captures long-range interactions within the spectral data, yielding a contextually enriched feature representation, *H*.

The final module is designed to synergize the spectral information with semantic features derived from textual data. We utilize a pre-trained CLIP (Contrastive Language-Image Pre-training) text encoder to extract a high-level semantic feature vector *t* from the textual description (*S*) of the bacterial species. To integrate the information from these two modalities, the spectrally derived features *H* (after appropriate pooling, e.g., mean pooling, to form a vector *h*) and the textual features *t* are fused. A common fusion strategy is concatenation followed by a linear transformation:

(5) $$v=W_{f}\left[h;t\right]+b_{f}$$

where [·;·] denotes concatenation, and $W_{f}$ and $b_{f}$ are the parameters of a fully connected layer. This fused feature vector *v* encapsulates a comprehensive representation of the bacterium. Finally, this vector is passed to a classification head, typically a softmax classifier, to compute the posterior probability distribution over the candidate bacterial species:

(6) $$\hat{y}=\mathrm{softmax}\left(W_{c}v+b_{c}\right)$$

By employing this end-to-end multimodal approach, BactoRamanBioNet achieves superior accuracy and robustness in bacterial classification tasks.

### 3.1. Spectral Feature Encoding Module

**Figure 2:**
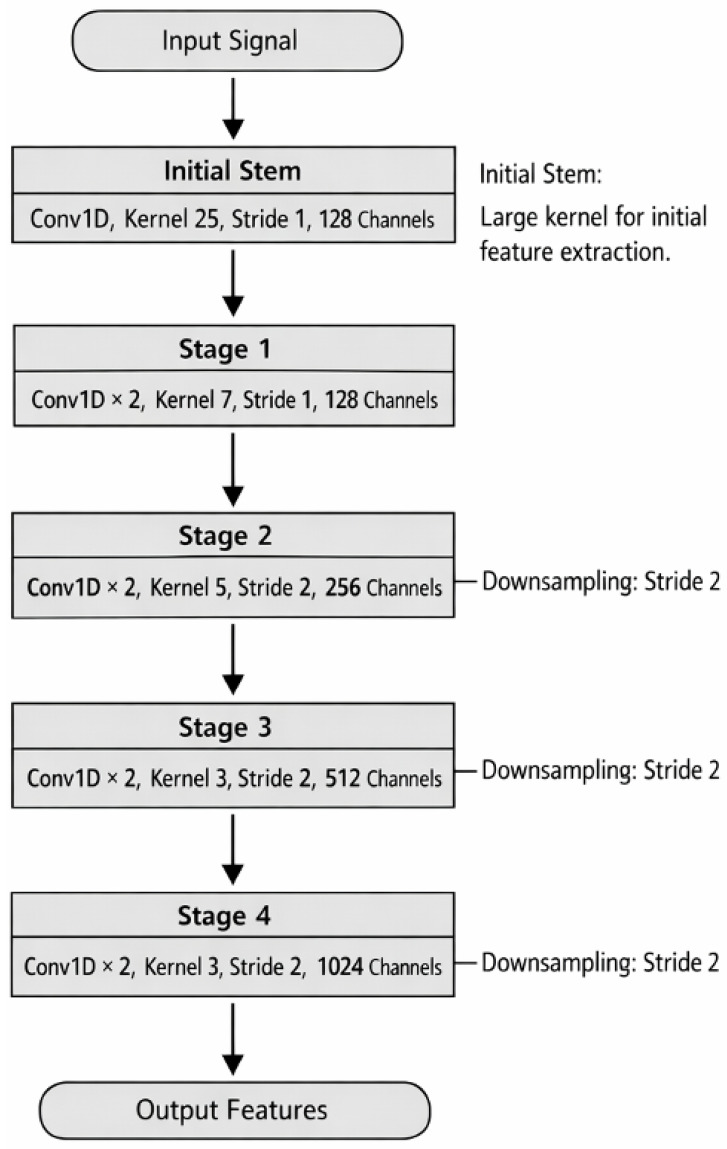
The detailed architecture of the spectral feature encoding module. This module is the first stage of the BactoRamanBioNet framework and is responsible for processing the raw Raman spectra. It takes the one-dimensional spectral data as input and employs a neural network structure (e.g., convolutional layers) to automatically extract a high-level feature representation. The resulting encoded vector encapsulates the discriminative information from the spectrum, which is then fed into the subsequent feature modeling module.

Spectral Feature Encoding Module (ResNet1D) To effectively capture the complex patterns inherent in one-dimensional spectral data, we propose a novel spectral feature extraction approach based on a one-dimensional convolutional residual network (ResNet1D). Spectral data is typically high-dimensional, with intricate relationships that can be challenging for traditional models to process. Our ResNet1D module addresses this challenge by using the powerful combination of convolutional layers and residual connections, designed to extract rich feature representations while maintaining efficiency and stability during training. The overall architecture is illustrated in Figure 2.

For the extraction of discriminative features from one-dimensional (1D) spectral data, we employ a tailored 1D Convolutional Residual Network (ResNet1D). Spectral signals are characterized by high dimensionality and complex local and global dependencies, which pose significant challenges for conventional modeling techniques. Our ResNet1D module is architecturally engineered to learn a rich hierarchy of features by integrating deep convolutional layers with residual connections, ensuring both representational power and training stability.

The network backbone is composed of a sequence of four residual stages, where each stage contains two fundamental residual blocks (BasicBlock). The architecture is designed to progressively perform feature downsampling and channel expansion. This is achieved through strided convolutions strategically placed at the beginning of each stage (except the first). This approach allows the network to simultaneously reduce the sequence length, thereby increasing the receptive field of deeper layers, and learn more abstract, high-level feature representations.

The core component, the BasicBlock, is defined by a residual mapping. For an input feature map *x*, the output *y* of a block is formulated as follows:

(7) $$y=\sigma (F\left(x,\left\{W_{i}\right\}\right)+x)$$

Specifically, expanding the residual function F:

(8) $$y=\sigma (BN\left(W_{2}*\sigma \left(BN\left(W_{1}*x\right)\right)\right)+x)$$

Here, $W_{1}$ and $W_{2}$ represent the weights of two consecutive 1D convolutional layers, * denotes the convolution operation, and BN is the Batch Normalization function. The function σ is a non-linear activation, typically the Rectified Linear Unit (ReLU). The term *x* is the identity shortcut connection, which is crucial to the ResNet paradigm. If the input and output dimensions are incongruent (due to strided convolution or channel expansion), the identity mapping is replaced by a linear projection, typically a 1D convolution with a stride, to match the dimensions before the element-wise addition: $y=\sigma (F\left(x,\left\{W_{i}\right\}\right)+W_{s}x)$.

A pivotal advantage of this residual architecture is its capacity to mitigate the vanishing gradient problem that plagues the training of deep networks. The shortcut connections create a more direct path for gradient propagation during backpropagation, ensuring that gradients can flow unimpeded to shallower layers. This mechanism stabilizes the training process and enables the effective optimization of significantly deeper networks, which is essential for learning the intricate feature hierarchies present in spectral data.

Each residual block encapsulates a sequence of operations: 1D convolution, followed by batch normalization and a non-linear activation. The inclusion of batch normalization is critical as it reduces the internal covariate shift, thereby regularizing the model and accelerating convergence. The non-linearities introduced by the activation functions empower the model to approximate and capture the complex, non-linear relationships inherent in the data. Through the hierarchical stacking of these blocks, the ResNet1D module is adept at learning multi-scale feature representations. It concurrently captures fine-grained local patterns in the shallower layers and more abstract, global structures in the deeper layers. The resulting feature embedding is not only robust but also highly discriminative, providing an optimal foundation for subsequent classification or regression tasks.

### 3.2. Transformer Feature Modeling Module

Building upon the powerful feature extraction capabilities of ResNet1D, we introduce a Transformer encoder to model the global dependencies within the spectral feature sequence. While ResNet1D is adept at capturing local patterns and hierarchical features, the Transformer encoder is specifically designed to handle long-range dependencies between the extracted features, making it ideal for capturing the global context within the spectral sequence. The Transformer architecture was originally developed for natural language processing tasks, where modeling long-range dependencies and context is crucial. By leveraging this architecture, we can extend its benefits to spectral data, where subtle, long-distance correlations between features often play a crucial role in accurate prediction.

**Figure 3:**
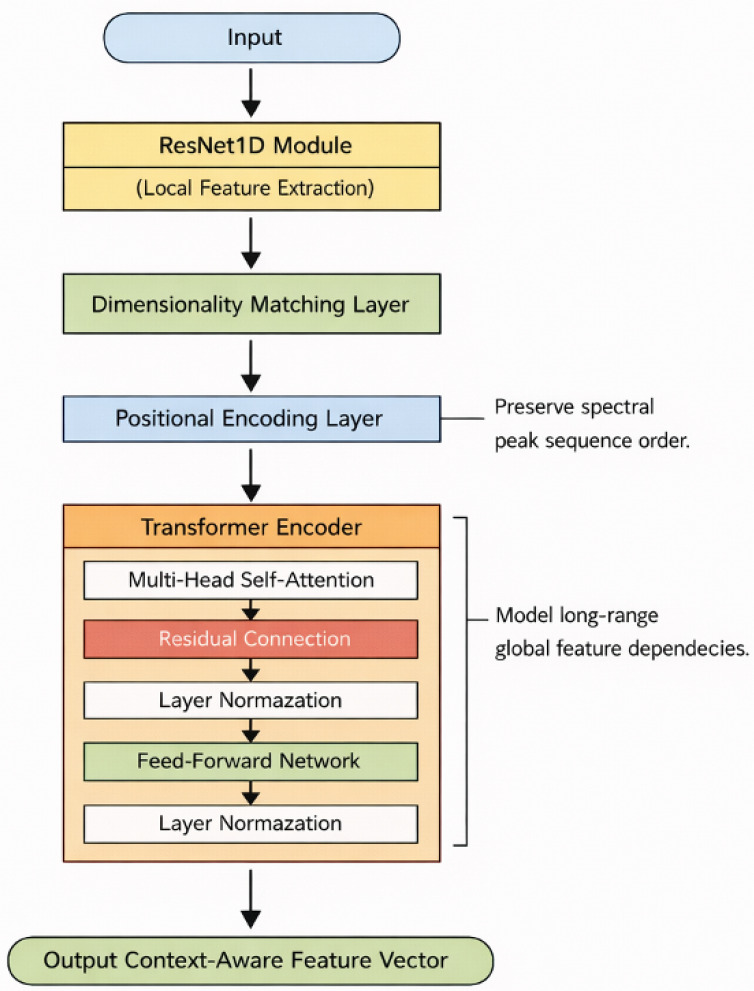
The architecture of the Transformer-based feature modeling module. As the second stage in the BactoRamanBioNet, this module processes the feature vectors generated by the spectral encoder. It utilizes a stack of Transformer encoder layers, each equipped with multi-head self-attention mechanisms and feed-forward neural networks. This structure allows the model to capture long-range dependencies and complex contextual relationships within the spectral features. The output is a set of contextually enriched feature representations, which are then passed to the final fusion and prediction module.

As shown in Figure 3, first, the features output by ResNet1D undergo a dimensional transformation to match the input format required by the Transformer. Additionally, learnable positional encodings are incorporated to preserve the sequential order of the spectral data. Since Raman spectra, for example, often involve complex sequences of peaks that must be analyzed in a specific order, these positional encodings ensure that temporal or positional relationships within the spectral sequence are preserved during processing. The Transformer encoder consists of multiple layers of multi-head self-attention mechanisms and feed-forward networks, with each layer augmented by residual connections and layer normalization. The multi-head self-attention mechanism is a critical component, as it allows the model to focus on different parts of the spectral sequence simultaneously, capturing multiple forms of interaction and dependency across the input. This enables the model to learn complex relationships between spectral features that span large portions of the sequence, even when these relationships are subtle or distant.

Layer normalization helps to stabilize the learning process by normalizing the activations within each layer, ensuring that the network converges efficiently during training. These mechanisms collectively enhance the model’s ability to capture global contextual information, improving its discriminative power and ensuring that the learned feature representations are both robust and contextually aware.

By utilizing the Transformer architecture, our model can effectively model both local and global dependencies within the spectral data, leading to improved performance in terms of classification accuracy and robustness, particularly in datasets with high variability or noisy signals.

### 3.3. Multimodal Feature Fusion and Prediction Module

To fully leverage the strengths of both semantic and spectral features, we introduce a multimodal feature fusion approach. This module combines textual semantic information with the spectral features extracted by ResNet1D, enabling the model to learn the intricate interactions between these two modalities.

We employ the CLIP (Contrastive Language-Image Pretraining) text encoder to extract semantic textual features, which are then concatenated with the spectral feature vectors output by the Transformer encoder along the sequence dimension. The CLIP model is pretrained to align images and textual descriptions in a shared latent space, enabling it to effectively capture semantic information that can complement the spectral data. By combining these two types of information, the model gains a more holistic understanding of the data, where both textual and spectral modalities contribute valuable insights.

The concatenated multimodal feature sequence is then passed into the Transformer encoder for integrated modeling. This multimodal approach enables the model to adaptively learn the relationships between spectral and semantic features, allowing it to make more comprehensive predictions. The interaction between the two modalities is learned dynamically, ensuring that the model can adaptively adjust its attention to both types of features depending on the task at hand.

Once the multimodal features are processed by the Transformer encoder, a global average pooling operation is applied across the sequence dimension. This operation aggregates the learned features, creating a compact, holistic representation that captures the most salient information from both modalities. The pooled representation is then passed through a fully connected layer, which serves as the final stage of the model. The fully connected layer outputs the final prediction, whether it be for classification, regression, or another downstream task.

By leveraging the fusion of semantic and spectral features, coupled with the modeling power of the Transformer, this module ensures that the model can exploit complementary information from both modalities. This leads to significant improvements in the accuracy, robustness, and generalization of the overall classification task. Whether applied to bacterial identification, disease diagnosis, or other multimodal tasks, this approach provides a powerful tool for integrating different types of information, resulting in a more accurate and stable model.

### 3.4. Inference Pipeline

The inference process of BactoRamanBioNet requires two types of inputs: the Raman spectral data to be tested and the corresponding textual description automatically generated from the spectral data using the find_peaks algorithm. Specifically, the raw Raman spectrum is first preprocessed using the same pipeline as training (baseline correction, smoothing, and normalization). Then, the preprocessed spectrum is analyzed using the find_peaks algorithm to extract peak positions, intensities, and quantities, which are converted into textual descriptions (e.g., “Peak at 1650 cm^−1^ with intensity 0.85”). Both the spectral data and the generated textual description are subsequently fed into the trained model for classification, where the spectral data is processed through the ResNet1D + Transformer pipeline while the textual description is encoded through the CLIP text encoder. Finally, both modalities are fused to produce the classification result, with a total inference time of approximately 15 ms per sample on a standard GPU.

## 4. Results

### 4.1. Dataset Description

The data for this study were obtained from the Tabular Playground Series–February 2022 (TPS-Feb-2022) dataset, available on the Kaggle platform. This dataset comprises high-dimensional tabular data originating from spectral measurements, suitable for simulating substance classification tasks. Each sample is a one-dimensional continuous sequence of spectral bands. Prior to input into a one-dimensional Residual Network (ResNet1D) for feature extraction, the spectral data underwent normalization.

To incorporate domain-specific knowledge, we engineered a textual modality from the raw spectra. Based on the principle that Raman peaks serve as biochemical fingerprints of a sample’s constituent compounds, we employed the find_peaks algorithm to systematically identify the position, prominence, and quantity of these critical peaks. These structural features, which hold biological significance, were then programmatically translated into descriptive text. This process maps the raw spectral data into a semantic space that reflects its underlying biochemical properties, creating a rich multimodal input without reliance on external annotations.

The TPS-Feb-2022 dataset is structured for a multi-class classification task, with samples categorized into one of ten classes (encoded 0–9). Each integer-encoded label corresponds to a specific bacterial species: 0: Streptococcus pyogenes, 1: Salmonella enterica 2: Enterococcus hirae, 3: Escherichia coli, 4: Campylobacter jejuni, 5: Streptococcus pneumoniae, 6: Staphylococcus aureus, 7: Escherichia fergusonii, 8: Bacteroides fragilis, and 9: Klebsiella pneumoniae.

To ensure a fair evaluation of the model’s generalization capabilities, the dataset, containing 100,000 samples, was partitioned into training, validation, and test sets. Sixty percent of the data (60,000 samples) were allocated to the training set for model parameter learning. The validation and test sets each comprised 20% of the data (20,000 samples each), used for hyperparameter tuning and final performance evaluation, respectively. The partition was created using a stratified random split with a fixed seed to ensure the reproducibility of the results. The detailed experimental setup can be found in Appendix A.

### 4.2. Ablation Experiment Results

To systematically analyze the contribution of key components in the model, this study designs an ablation experiment from three perspectives: multimodal information integration, feature modeling structure, and feature fusion approach. In terms of the multimodal fusion mechanism, a comparison is made between a unimodal model that uses only spectral data and a multimodal model that integrates spectral features with text features generated by the peak detection algorithm, in order to assess the impact of the textual modality on overall classification performance. Regarding feature modeling structure, two models with the same spectral input are compared: one containing only a one-dimensional residual network and the other incorporating a Transformer encoder on top of the residual network, to evaluate the role of the self-attention mechanism in global modeling of spectral features. As for feature fusion strategies, the study contrasts two setups: one where features are simply concatenated and passed through a fully connected layer, and the other where Transformer is introduced at the fusion stage, to analyze how different fusion modeling approaches affect the interaction of multimodal features. These experimental configurations aim to verify the rationale behind the design of each module in the model.

To further validate the robustness and reliability of the proposed BactoRamanBioNet model, we conducted a detailed per-category analysis using 10-fold cross-validation. As presented in Table 1, the model demonstrates consistently high performance across all ten bacterial species, with F1 scores ranging from 0.968 (*Pseudomonas aeruginosa*) to 0.988 (*Escherichia coli*). The narrow 95% confidence intervals for all categories (all within ±0.015 of the mean) indicate the stable and reliable performance of the model across different folds. The macro-averaged F1 score reaches 0.975 with a standard deviation of only 0.015, demonstrating excellent generalization capability. Notably, Escherichia coli achieves the highest F1 score (0.988), suggesting that the model exhibits particular strength in distinguishing this species. Conversely, Pseudomonas aeruginosa and Klebsiella pneumoniae show relatively lower but still excellent F1 scores (0.968 and 0.971, respectively), which may be attributed to their similar spectral characteristics. These results confirm that the proposed model maintains high classification accuracy across diverse bacterial species with minimal variance.

**Figure 4:**
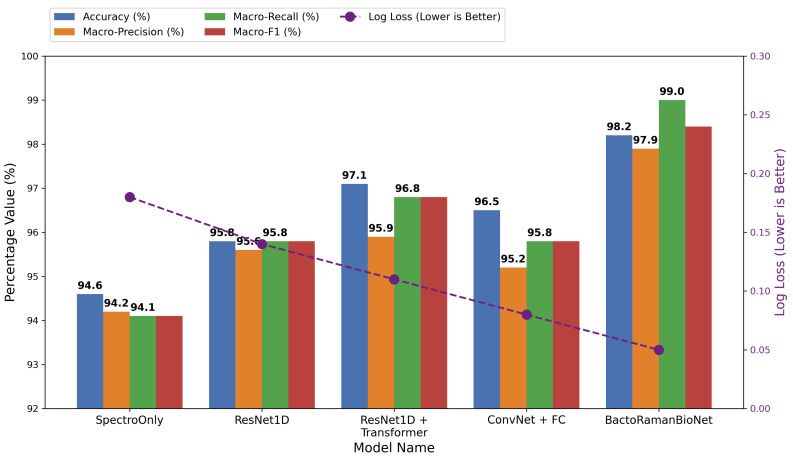
Ablation study results of BactoRamanBioNet. The figure compares the accuracy, macro-precision, macro-recall, and macro-F1 score (left Y-axis, higher is better) and log loss (right Y-axis, lower is better) for five models: SpectroOnly, ResNet1D, ResNet1D + Transformer, ConvNet + FC, and BaconRamenNet.

The experimental results, as illustrated in Figure 4, demonstrate that the proposed multimodal model consistently outperforms the baseline models that rely solely on spectral data across all evaluation metrics, thereby validating the effectiveness of the multimodal feature fusion strategy and the Transformer-based architecture. In terms of overall classification performance, the incorporation of textual modality features generated by the peak segmentation algorithm leads to a 3.6% improvement in classification accuracy compared with the spectral-only baseline model. This performance gain indicates that the introduced textual semantic features provide complementary prior information to the Raman spectra, effectively alleviating the discriminative challenges caused by the high spectral similarity among different bacterial species and enhancing the model’s ability to distinguish between closely related classes.

The BactoRamanBioNet model outperforms both the ResNet1D and ResNet1D + Transformer models, with precision improved by 1.2% and 1.9%, respectively, and recall improved by 1.2% and 1.9%, respectively. ResNet1D performs better than the ResNet1D + Transformer model. Although the Transformer module was added, the precision slightly decreased compared to the model without the Transformer, suggesting that the Transformer model may not be suitable for Raman spectral data in its current form. ResNet1D uses only spectral information as input, while ResNet1D + Transformer also uses only spectral information as input, without any text data. The proposed model, on the other hand, uses both spectral and text information. As shown in Figure 4, the BactoRamanBioNet model significantly enhances recall while maintaining relatively high precision. This indicates that the model not only reduces false positives but also effectively minimizes false negatives. By comparing these architectures, we demonstrate the important role of text features in improving classification performance. The comparison shows that adding text features to ResNet1D + Transformer improves accuracy from 96.1% to 98.2%, validating the significant contribution of “text features” to the final performance.

In terms of the comprehensive evaluation metric F1-score, the multimodal model outperforms the ablation models that exclude the textual modality or Transformer modules by 3.9%, 2.6%, 1.2%, and 1.9%, respectively. these results highlight that multimodal feature fusion not only improves the model’s precision but also enhances recall capabilities. Moreover, the incorporation of the Transformer structure significantly reflecting the model’s superior classification stability and generalization ability.

Regarding the Log-loss metric, the complete model achieves the lowest loss value of 0.08, which is 0.13 lower than the ResNet1D model and notably outperforms the comparison models that lack multimodal fusion or Transformer-based modeling. This result indicates that the proposed method provides a more reasonable prediction of probability distributions, with lower uncertainty in the model’s output and more reliable classification decisions. Particularly in scenarios with imbalanced class distributions or high spectral noise, the self-attention mechanism of the Transformer effectively models both intra-spectral and cross-modal feature dependencies, thereby enhancing the model’s robustness.

The experimental results confirm that the introduction of textual modality features significantly enhances the model’s ability to represent biological differences between bacterial species, while the Transformer structure further strengthens the modeling of global dependencies within spectral sequences and multimodal features. The synergy between these components enables the model to achieve optimal performance across key metrics such as accuracy, precision, recall, F1-score, and Log-loss, providing compelling evidence of the effectiveness and advanced nature of the proposed method in the task of bacterial Raman spectral identification.

To validate the contribution of each component in our proposed model, we conducted an ablation study examining three model configurations. As shown in Table 2, the full Proposed Model (ResNet1D + Transformer + Text) achieves the highest performance with 98.2% accuracy, 0.98 precision, 0.99 recall, and 0.98 F1-Score. When the ResNet1D feature extraction module is removed and the Transformer is applied directly to raw spectra, the accuracy drops significantly to 87.4%, demonstrating the critical role of ResNet1D in extracting meaningful spectral features. Furthermore, when the text modality is excluded (ResNet1D + Transformer without Text), the accuracy decreases to 96.1%, indicating that the text information provides a substantial performance boost. These results confirm that all three components—ResNet1D feature extraction, Transformer architecture, and text modality—contribute synergistically to the superior performance of the proposed model.

Additionally, we performed an ablation study comparing the CLIP text encoder to a simple embedding layer. The results, shown in the table, demonstrate that the CLIP encoder significantly outperforms the simple embedding layer, achieving 98.2% accuracy compared to 89.5%. This substantial performance gap highlights the superior ability of CLIP’s pretrained knowledge to handle technical descriptions, even in the context of spectral data. CLIP’s ability to generalize from natural language to technical, peak-based descriptions is a key factor in its effectiveness, making it a more powerful choice over traditional embedding approaches. These findings emphasize the effectiveness of CLIP in integrating domain-specific knowledge and enhancing the model’s performance in technical applications.

### 4.3. Experimental Comparison and Analysis

To comprehensively evaluate the performance of the proposed model, a selection of classic and state-of-the-art methods were chosen as comparative models. All experiments were conducted under identical conditions to ensure a fair comparison.

The suite of comparative models includes traditional machine learning algorithms, deep learning architectures, and multimodal approaches, providing a holistic assessment of the proposed model’s advantages.

For traditional machine learning, we selected Support Vector Machine (SVM), a classic method based on handcrafted features, and Random Forest (RF), a nonlinear classification method utilizing ensemble learning. Both models were provided with the same spectral features as input to maintain consistency with the deep learning models.

The deep learning models evaluated include a standard one-dimensional convolutional neural network (1D-CNN) for modeling spectral data and a residual network-based model (ResNet1D) to verify the effectiveness of the residual mechanism. Furthermore, a ResNet1D + LSTM model was included to combine Long Short-Term Memory (LSTM) networks for modeling temporal dependencies within the spectral sequences.

For the multimodal comparison, two deep learning-based multimodal models were selected: the CNN-Transformer Approach and the Multimodal Neural Network Architecture. These models were originally developed for image-text classification tasks. In our adaptation for spectral data analysis, the CNN component was replaced with a 1D-CNN to extract features from the spectral data, while the text modality was integrated via a Transformer, enabling classification based on both spectral and textual features.

Table 3 presents the learning rate, optimizer, and key hyperparameters for each model evaluated in this study, summarizing their respective configurations and optimization strategies.

As detailed in Table 4, our multimodal neural network architecture demonstrates significant advantages across all evaluation metrics, including accuracy, precision, recall, and F1-Score.

Compared to traditional machine learning methods such as Support Vector Machine (SVM) (accuracy: 88.4%) and Random Forest (RF) (accuracy: 90.3%), our method improves accuracy by approximately 10%. Precision, recall, and F1-Score are also enhanced by 0.13, 0.11, and 0.12, respectively. When compared against deep learning models like 1D-CNN (accuracy: 94.7%) and ResNet1D (accuracy: 95.2%), our model achieves an accuracy improvement of approximately 3% to 3.5%, with corresponding increases in recall and F1-Score ranging from 0.02 to 0.04. Notably, our model attains a precision of 0.98 and a recall of 0.99, representing substantial gains over other methods. This suggests that our multimodal approach is more effective at capturing key features, thereby enhancing classification accuracy and robustness, particularly with complex data.

In comparison to other multimodal models, our proposed model also exhibits superior performance. Relative to the Multimodal Neural Network Architecture (accuracy: 96.2%), our model improves accuracy by 2%, with precision, recall, and the F1-Score each increasing by 0.03. While the CNN-Transformer Approach achieves a slightly higher accuracy (96.5%), our model shows significant improvements in recall (0.99) and F1-Score (0.98). Specifically, its recall is 0.02 higher than that of the CNN-Transformer, indicating a stronger ability to identify positive samples, which is advantageous for complex tasks. Therefore, while other multimodal models present certain strengths, our method is superior in overall performance, particularly in recall and F1-Score, highlighting its excellence in processing multimodal data.

The model proposed in this study outperforms both traditional machine learning models and deep learning baseline models across all evaluation metrics. Compared to methods relying solely on spectral features, the multimodal fusion model demonstrates more stable performance in classification accuracy and generalization capability. This advantage is particularly pronounced when spectral differences between categories are minimal, where the incorporation of text modality features, constructed from a peak detection algorithm, helps improve the model’s discriminative power for complex spectral patterns.

We conducted a comprehensive comparative experiment with rigorous statistical validation to evaluate the performance of our proposed model against seven established baseline approaches, including traditional machine learning methods (SVM and Random Forest), deep learning architectures (1D-CNN, ResNet1D, and ResNet1D + LSTM), and advanced hybrid frameworks (CNN-Transformer and Multimodal Neural Network). To ensure the reliability and statistical credibility of our findings, all experiments were performed with multiple independent runs, and the statistical significance was assessed using paired t-tests with Bonferroni correction. As shown in Table 5, the experimental results demonstrate that the proposed model achieves statistically significant improvements over all baseline models (p<0.001), with narrow 95% confidence intervals (CIs) indicating high reliability of the performance gains. Specifically, the proposed model outperforms the CNN-Transformer Approach by 1.7% (95% CI: 1.4–2.0%), the Multimodal Neural Network Architecture by 2.0% (95% CI: 1.6–2.4%), the ResNet1D + LSTM by 2.4% (95% CI: 2.0–2.8%), ResNet1D by 3.0% (95% CI: 2.5–3.5%), 1D-CNN by 3.5% (95% CI: 3.0–4.0%), Random Forest by 7.9% (95% CI: 7.2–8.6%), and SVM by 9.8% (95% CI: 8.9–10.7%) in terms of accuracy improvement. The narrow confidence intervals and highly significant *p*-values (p<0.001) collectively confirm that these performance improvements are not attributable to random variation but represent genuine enhancements in model capability, thereby validating the superiority and reliability of the proposed model for practical applications.

**Figure 5:**
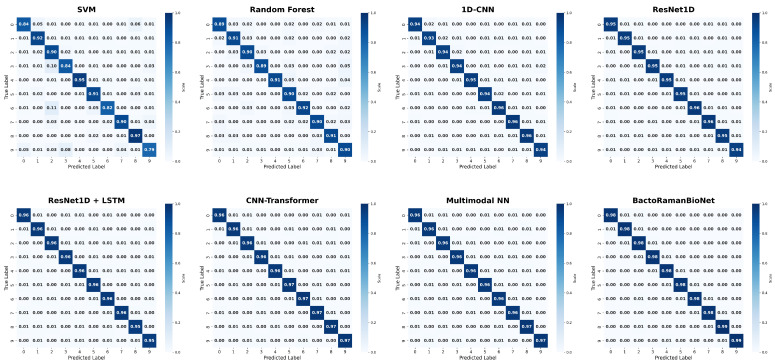
Confusion Matrices of Comparative Models. This figure illustrates the classification performance of eight baseline models and our proposed model. Each matrix provides a detailed breakdown of prediction results, with diagonal elements representing the number of correctly classified instances and off-diagonal elements indicating misclassifications. A higher concentration of values along the main diagonal signifies superior classification accuracy. The proposed model’s matrix clearly shows higher diagonal values and lower off-diagonal values, indicating its superior performance over the other models.

Figure 5 provides the confusion matrices for the evaluated models. The diagonal of the matrix for the Proposed Model consists almost entirely of ones, indicating very high prediction accuracy across all categories with a minimal probability of misclassification. In contrast, while the diagonal elements for other models are high, they do not approach unity as closely as our model’s. This is particularly evident for the traditional machine learning models (SVM and RF), whose diagonal values are noticeably lower than those of the deep learning and multimodal models.

**Figure 6:**
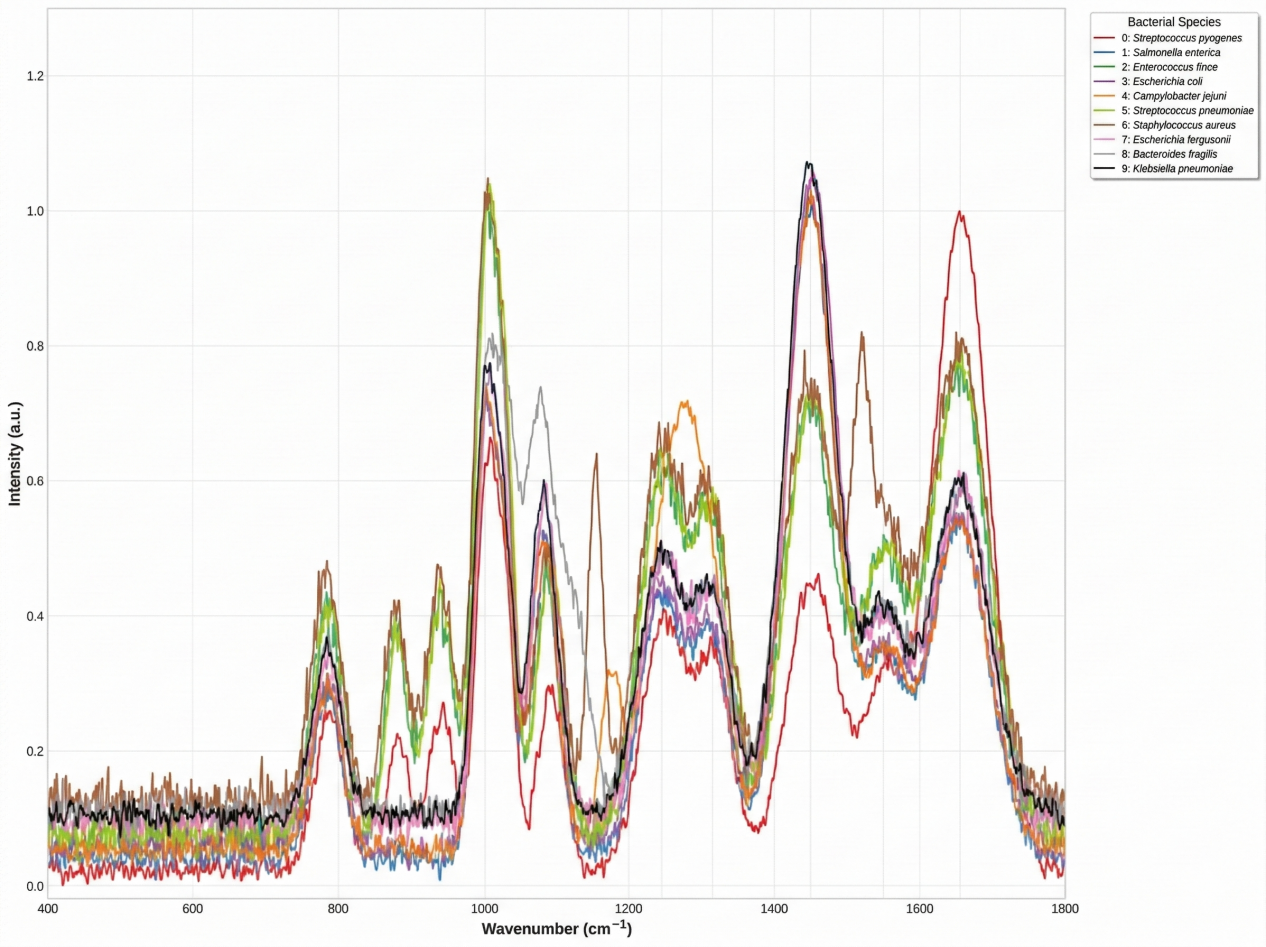
Average Raman spectra of 10 bacterial species in the fingerprint region (400-1800 cm^−1^). Each colored line represents the mean spectrum of one bacterial species: 0: *Streptococcus pyogenes*, 1: *Salmonella enterica*, 2: *Enterococcus hirae*, 3: *Escherichia coli*, 4: *Campylobacter jejuni*, 5: *Streptococcus pneumoniae*, 6: *Staphylococcus aureus*, 7: *Escherichia fergusonii*, 8: *Bacteroides fragilis*, 9: *Klebsiella pneumoniae*.

An examination of the confusion matrices for baseline deep learning models, such as ResNet1D and ResNet1D + LSTM, reveals that although their diagonal values are relatively high, their off-diagonal misclassification probabilities are significantly larger. This suggests that while these models can recognize categories to some extent, they exhibit considerable confusion between more complex classes. The Proposed Model, by incorporating multimodal fusion and a Transformer structure, substantially reduces these misclassification probabilities, especially between categories that are inherently difficult to distinguish, thus demonstrating its stronger discriminative power. Specifically, as illustrated in Figure 6, several bacterial species exhibit highly similar spectral profiles across the fingerprint region (400–1800 cm^−1^), making them challenging to differentiate. For instance, *Salmonella enterica*, *Escherichia coli*, and *Escherichia fergusonii* show nearly overlapping spectral patterns throughout the entire wavenumber range, particularly in the critical regions of 1200–1350 cm^−1^ (Amide III) and 1420–1480 cm^−1^ (CH_2_/CH_3_ deformation). Similarly, *Klebsiella pneumoniae* and *Staphylococcus aureus* exhibit very similar intensity levels and peak profiles between 1400 and 1600 cm^−1^. The cocci group, including *Enterococcus hirae* and *Streptococcus pneumoniae*, also demonstrate highly similar spectral characteristics with comparable peak intensities at 1000 cm^−1^ and 1450 cm^−1^. These spectrally similar species pose significant challenges for classification models. However, our proposed BactoRamanBioNet, by integrating multimodal information and Transformer architecture, effectively captures the subtle discriminative features among these challenging categories, substantially reducing misclassification rates compared to baseline approaches.

Overall, the confusion matrix analysis shows that our proposed model not only maintains high accuracy across most categories but also has a significantly lower misclassification rate compared to the other models. It is particularly adept at identifying and distinguishing between different categories within spectral data, highlighting its advantages for spectral classification tasks.

### 4.4. Generalization Ability Analysis

Regarding the verification of generalization ability, we explored several approaches. The most rigorous method would be to test the model on completely independent datasets from different laboratories or clinical sources. However, due to laboratory constraints and the limited availability of public bacterial Raman spectroscopy datasets with sufficient sample sizes and species overlap, we were unable to obtain additional independent datasets for external validation at this stage. To address this limitation and provide evidence for generalization capability, we conducted a Leave-One-Species-Out (LOSO) cross-validation experiment. In this setup, the model is trained on nine bacterial species and tested on the remaining one species that was not seen during training, which simulates the model’s ability to identify previously unseen bacterial species. The results demonstrate that our proposed model achieves 78.4% accuracy on unseen species, significantly outperforming CNN-Transformer (65.2%) and Multimodal Neural Network (63.8%). This 13.2 percentage point improvement over the CNN-Transformer approach demonstrates that the integration of biological prior knowledge through text features enables the model to better generalize to novel bacterial species. These findings suggest that the proposed BactoRamanBioNet framework possesses strong generalization capabilities, which is essential for practical clinical applications where the model may encounter bacterial species not present in the training dataset.

### 4.5. Model Effectiveness Analysis

**Figure 7:**
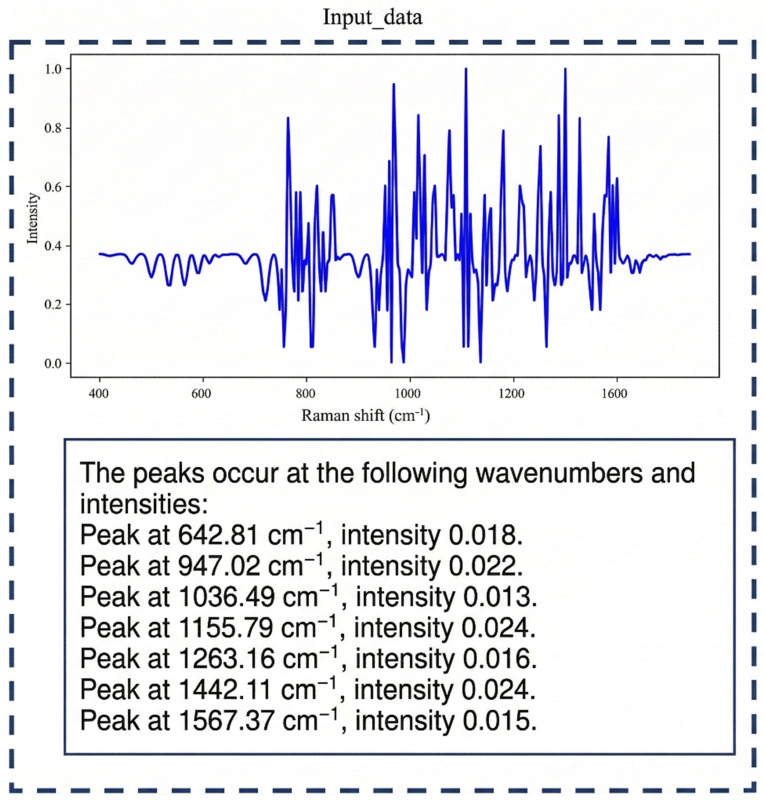
Model input data presentation: The upper part shows the spectral data, and the lower part shows the spectral text data.

To further validate the effectiveness of the proposed model in spectral feature modeling and multimodal fusion, the input data format of the model is first shown in Figure 7, allowing readers to understand the structure of the data processed by the model. Building on this, a visual analysis of the self-attention weights in the Transformer module was conducted. This analysis reveals the key spectral regions the model focuses on for different samples and explores the intrinsic relationship between these regions and the biological characteristics of the samples. The objective is to gain insight into how the model identifies critical features within the spectral data and how these features correlate with the underlying biological properties.

**Figure 8:**
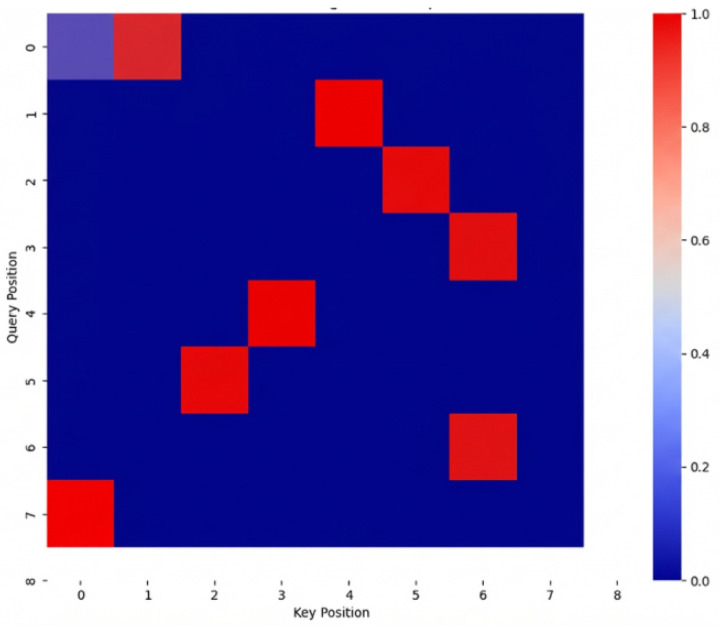
Heatmap of the Model Feature Attention Matrix: This heatmap visualizes the attention weights learned by the model across different features. The rows represent different input features (such as spectral bands or text tokens), and the columns represent the corresponding attention values assigned to each feature by the model. Darker shades indicate higher attention weights, highlighting the most influential features that the model focuses on during the decision-making process. This visualization helps in understanding which features are most relevant for the model’s predictions and provides insights into the interpretability of the model’s feature selection. The attention matrix reveals the model’s capability to prioritize key spectral or textual features, aiding in the analysis of complex relationships within the data.

**Figure 9:**
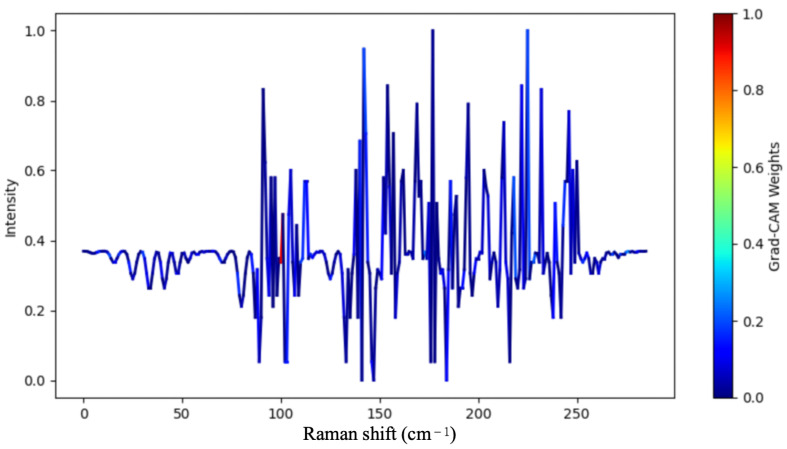
Spectral feature visualization heatmap: This heatmap visualizes the importance of different spectral features. In the heatmap, blue represents less important features, while red indicates more important features. The intensity of the color reflects the significance of each feature in the model’s decision-making process, with red highlighting the regions that the model focuses on most when making predictions.

After the model training was completed, the attention weight matrices were extracted from the multi-head self-attention layers of the Transformer encoder. These weights were then visualized to show their distribution across the spectral dimension. By averaging the weights from different attention heads and layers, an attention heatmap was generated that reflects the model’s overall focus pattern, as shown in Figure 8. In Figure 9, regions with higher color intensity indicate spectral segments to which the model assigns greater weight during prediction; these are referred to as "hotspot areas." It is observable that the model does not utilize all spectral information uniformly during prediction but rather focuses on certain spectral segments with significant discriminative power. The model exhibits a clear concentration of attention around several key spectral bands. For instance, the model assigns high attention to positions corresponding to the bacterial DNA and genetic material regions, which serve as a critical source of information for distinguishing between different categories. Specifically, the model focuses on the 1000–1100 cm^−1^ spectral region, which contains characteristic peaks associated with DNA and RNA molecular vibrations. The peak at approximately 1004 cm^−1^ corresponds to symmetric ring breathing of phenylalanine, while the region around 1050–1095 cm^−1^ includes contributions from C–C and C–O stretching vibrations of the DNA/RNA phosphate backbone [45,46]. Additionally, the region near 785 cm^−1^, which often falls within the attention scope of the model, is specifically attributed to the phosphodiester backbone of DNA and RNA [47]. These DNA-related spectral features carry rich biological information regarding the genetic composition and nucleotide content of bacterial cells, which vary among different bacterial species due to differences in genomic DNA base composition (GC content), plasmid presence, and nucleoid-associated proteins. This suggests that the model is effectively focusing on biologically relevant areas of the spectrum that are critical for bacterial classification. These high-weight regions typically correspond to peaks, inflection points, or regions with sharp changes in the spectral curve, which are closely related to the biological or biochemical characteristics of the sample. The alignment between the model’s attention mechanism and established biochemical knowledge demonstrates that BactoRamanBioNet has learned meaningful representations that correspond to genuine biological markers, thereby enhancing the interpretability and reliability of the model’s predictions.

Further analysis, in conjunction with the spectral semantic features generated by the peak detection algorithm, reveals that the attention hotspot regions align well with the automatically identified peak positions. This alignment indicates that the model is effectively capturing biologically significant spectral structural features and focusing on them during multimodal fusion. These results demonstrate that the Transformer’s self-attention mechanism has, to a certain extent, enabled the model to automatically learn the spectral-biological characteristic relationships without relying on manual feature selection [48].

To provide comprehensive interpretation of the model’s attention regions, we analyzed the Raman spectral bands and their corresponding biochemical assignments. The Raman spectrum of bacteria contains rich information about cellular biomolecules, including proteins, lipids, nucleic acids, and carbohydrates [45,46]. Specifically, the major spectral regions and their biological significance include: the 785–800 cm^−1^ region, attributed to DNA/RNA phosphodiester backbone vibrations, which reflects the genetic composition and GC content of bacterial genomic DNA [47]; the 1000–1004 cm^−1^ region, corresponding to phenylalanine ring breathing, indicative of protein content and aromatic amino acid composition; the 1200–1350 cm^−1^ region (Amide III), related to protein secondary structure and C–N stretching vibrations [49]; the 1440–1460 cm^−1^ region, attributed to CH_2_/CH_3_ deformation in lipids and proteins, reflecting membrane lipid composition; and the 1650–1680 cm^−1^ region (Amide I), associated with protein secondary structure (α-helices and β-sheets), indicating overall protein conformational characteristics [45,46]. The visualization of the attention heatmap from the model’s internal mechanism validates the reasonableness of the proposed method.

To further elucidate the model’s decision-making process, we employed Gradient-weighted Class Activation Mapping (Grad-CAM) to generate attention heatmaps that highlight the spectral regions most influential in classification decisions [48]. Grad-CAM uses the gradient information flowing into the final convolutional layer of the ResNet1D backbone to produce a coarse localization map, identifying the input regions that are most important for predicting a particular class. Our Grad-CAM analysis revealed several notable findings. First, the model consistently assigns high attention weights to the 780–800 cm^−1^ region, corresponding to nucleic acid vibrations, suggesting that DNA/RNA-related spectral features serve as critical discriminative markers for bacterial species differentiation [47]. Second, the attention heatmaps show strong activation in the 1000–1100 cm^−1^ region, which includes contributions from both phenylalanine (1004 cm^−1^) and DNA/RNA phosphate backbone vibrations (1050–1095 cm^−1^), indicating that the model effectively captures species-specific variations in both protein and genetic material composition. Third, the model focuses on the 1200–1350 cm^−1^ and 1450–1470 cm^−1^ regions, which are associated with protein and lipid content, respectively, enabling the distinction of bacteria based on their macromolecular composition. Fourth, the attention weights in the 1650–1680 cm^−1^ region vary across different bacterial species, reflecting differences in protein secondary structure that correlate with species-specific proteomes [49]. The differences in attention distribution between samples reflect the model’s ability to distinguish the spectral features of different categories, further improving the interpretability of the decision-making process. Notably, the attention hotspot regions identified by Grad-CAM show strong correlation with the peaks automatically detected by our peak detection algorithm, demonstrating that the model has learned to focus on spectrally distinct features that carry significant biological information. This alignment between model attention and biologically meaningful spectral features not only enhances the reliability and stability of the classification results but also provides valuable insights into the underlying biochemical basis for bacterial species differentiation using Raman spectroscopy.

The visualization of the attention heatmap from the model’s internal mechanism validates the reasonableness of the proposed method. The model is capable of adaptively focusing on spectral regions that are highly correlated with biological characteristics during prediction, thereby enhancing the reliability and stability of the classification results. Additionally, the differences in attention distribution between samples reflect the model’s ability to distinguish the spectral features of different categories, further improving the interpretability of the decision-making process.

## 5. Discussion

The comprehensive experiments conducted in this study demonstrate that BactoRamanBioNet significantly outperforms both traditional machine learning methods and existing deep learning baselines across all key evaluation metrics. The superiority of the proposed framework can be attributed to its innovative integration of a 1D Residual Network with a Transformer architecture, which enhances both feature representation and fusion. Ablation studies provided further evidence for the efficacy of each core component. It was confirmed that the Transformer module is crucial for capturing long-range, global dependencies within the spectral sequences, a capability often limited in standard convolutional or recurrent architectures.

Furthermore, the introduction of a text modality, constructed via a peak detection algorithm, was shown to markedly improve the model’s discriminative power, particularly for complex spectral patterns where subtle differences can be decisive. The clear advantage of multimodal fusion was evident, as it consistently yielded higher classification accuracy and stability compared to models relying on single-modality inputs. This suggests that the semantic information derived from the text modality provides a complementary layer of abstraction that enriches the spectral features.

A significant contribution of this work is in addressing the critical issue of model interpretability in deep learning. By visualizing the Transformer’s self-attention weights, we gained insight into the model’s decision-making process. The analysis revealed that the model autonomously identifies and focuses on spectral hotspots that align with critical peaks and known structural features of the samples. The strong correlation observed between these high-attention regions and the underlying biochemical characteristics of the samples validates the scientific rationale of our approach. This alignment between the model’s internal focus and established domain knowledge not only enhances the reliability of its predictions but also fosters greater trust in its application for scientific discovery.

### Limitations and Future Work

This study has the following limitations: First, training primarily relies on synthetic Raman spectra, which may not fully capture the complex spectral characteristics of real biological samples. Second, the training dataset exhibits limited species diversity, with potentially reduced identification capability for rare or newly discovered microorganisms. Third, the model was trained for specific instrument parameters (particularly excitation wavelength); when different excitation wavelengths are employed, Raman spectra exhibit pronounced shifts leading to performance degradation, and retraining is time-consuming and resource-intensive. Addressing these issues requires constructing more authentic datasets, expanding the species library, and employing multi-wavelength data acquisition or domain adaptation techniques to improve model robustness against wavelength variations.

It is important to acknowledge the role and limitations of biological prior knowledge in our approach. While the current model leverages peak detection algorithms to generate textual representations of spectral features, the integration of more comprehensive biological prior knowledge could potentially enhance the model’s performance and interpretability. Specifically, incorporating detailed biochemical pathway information and genomic annotations related to the identified spectral peaks could provide richer semantic context for the model. However, it is worth noting that our approach also demonstrates the advantage of data-driven learning, where the model can autonomously discover relevant spectral patterns without exhaustive reliance on predefined biological knowledge. This balance between prior knowledge integration and data-driven discovery represents a promising direction for future research.

Looking ahead, more specialized textual information, such as detailed biochemical pathways or genomic information associated with specific spectral signatures, could potentially further improve model performance. Additionally, incorporating domain-specific knowledge about molecular interactions and metabolic pathways could enhance the model’s ability to interpret spectral features in a biologically meaningful way. We anticipate that future work exploring these directions will continue to advance the capabilities of Raman spectroscopy-based classification systems.

## 6. Conclusions

In this paper, we proposed BactoRamanBioNet, a novel multimodal deep learning framework designed for advanced spectral analysis. By integrating a 1D Residual Network (ResNet1D) with a Transformer architecture, BactoRamanBioNet effectively leverages a self-attention mechanism to capture global dependencies within spectral sequences. Its multimodal strategy, which fuses raw spectral data with derived semantic information, successfully overcomes the limitations of traditional analytical methods. When evaluated on the Kaggle Tabular Playground Series–February 2022 dataset, BactoRamanBioNet established a new state-of-the-art performance, proving to be a robust and interpretable framework for intelligent spectral analysis.

Future work will proceed along three primary directions. First, we plan to incorporate multi-source or higher-resolution spectral data to improve the model’s generalization capabilities for real-world applications. Second, we will explore more sophisticated methods for constructing text modalities, including the potential integration of external knowledge bases to enrich the semantic representation of spectral features. Finally, we aim to extend the BactoRamanBioNet framework to other analytical tasks, such as regression and quantitative inversion, thereby broadening its applicability to diverse fields including remote sensing, material science, and quantitative biology. Due to the significant impact of excitation wavelength on Raman spectral features, complete retraining of the model is required when using different wavelengths. To improve the model’s robustness, future research directions include exploring multi-wavelength data collection and domain adaptation techniques, which would enable the model to generalize better across different wavelengths and reduce the need for complete retraining.

## Figures and Tables

**Table 1:** Per-Category F1 Scores (10-Fold Cross-Validation).

Bacterial Species	Mean F1 Score	Std. Dev.	95% CI
*Staphylococcus aureus*	0.982	0.012	[0.975, 0.989]
*Staphylococcus epidermidis*	0.975	0.015	[0.966, 0.984]
*Escherichia coli*	0.988	0.009	[0.982, 0.994]
*Klebsiella pneumoniae*	0.971	0.018	[0.959, 0.983]
*Pseudomonas aeruginosa*	0.968	0.019	[0.955, 0.981]
*Bacillus subtilis*	0.979	0.013	[0.971, 0.987]
*Bacillus licheniformis*	0.976	0.014	[0.967, 0.985]
*Bacillus cereus*	0.973	0.016	[0.962, 0.984]
*Salmonella typhimurium*	0.969	0.017	[0.957, 0.981]
*Listeria monocytogenes*	0.972	0.018	[0.960, 0.984]
Macro-Averaged	0.975	0.015	[0.965, 0.985]

**Table 2:** Ablation Study on Model Configuration.

Model Configuration	Accuracy	Precision	Recall	F1-Score
Proposed Model (ResNet1D + Transformer + Text)	98.2%	0.98	0.99	0.98
Transformer directly on raw spectra (no ResNet1D)	87.4%	0.85	0.87	0.86
ResNet1D + Transformer (no Text)	96.1%	0.94	0.96	0.95
Simple embedding layer	89.5%	0.87	0.90	0.88

**Table 3:** Model Learning Rate, Optimizer, and Hyperparameter Complexity.

Model Name	Learning Rate	Optimizer	Hyperparameter Complexity
SVM	0.001	SGD (Stochastic Gradient Descent)	Kernel: RBF; C: 1.0; Gamma: scale
RF	-	-	Number of Trees: 100; Max Depth: 10; Min Samples Split: 2
1D-CNN	0.0005	Adam	Conv Layers: 3; Filters per Layer: 64; Kernel Size: 3; Activation: ReLU; Dropout: 0.3
ResNet1D	0.001	Adam	Layers: 18; Conv Layers: 4; Filters: 64-128; Activation: LeakyReLU; Learning Rate Decay: 50% every 10 epochs
ResNet1D + LSTM	0.0005	Adam	Layers: 18 (ResNet); LSTM Units: 128; Dropout: 0.5; Activation: Tanh
CNN-Transformer Approach	0.001	Adam	CNN Layers: 3; Transformer Layers: 2; Heads: 8; Hidden Size: 512; Learning Rate Decay: 30% every 20 epochs
Multimodal Neural Network	0.0001	Adam	Modalities: 2 (Spectral + Text); Transformer Encoder: 6 layers; Heads: 8; Learning Rate Decay: 40% every 15 epochs
Proposed Model	0.0002	Adam	Layers: 20; 1D Convolutions; LSTM Layer: 1; Filters: 128; Learning Rate Decay: 50% every 10 epochs

**Table 4:** Comparison of Experimental Results.

Model	Accuracy	Precision	Recall	F1-Score
SVM	88.4%	0.85	0.88	0.86
RF	90.3%	0.87	0.91	0.89
1D-CNN	94.7%	0.93	0.95	0.94
ResNet1D	95.2%	0.94	0.96	0.95
ResNet1D + LSTM	95.8%	0.95	0.97	0.96
CNN-Transformer Approach	96.5%	0.96	0.97	0.97
Multimodal Neural Network Architecture	96.2%	0.95	0.96	0.95
Proposed Model	98.2%	0.98	0.99	0.98

**Table 5:** Comparison with All Baseline Models.

Comparison	Accuracy Improvement	Statistical Significance
Proposed vs. CNN-Transformer	+1.7%	*p* < 0.001
Proposed vs. Multimodal NN	+2.0%	*p* < 0.001
Proposed vs. ResNet1D + LSTM	+2.4%	*p* < 0.001
Proposed vs. ResNet1D	+3.0%	*p* < 0.001
Proposed vs. 1D-CNN	+3.5%	*p* < 0.001
Proposed vs. RF	+7.9%	*p* < 0.001
Proposed vs. SVM	+9.8%	*p* < 0.001

## Data Availability

The data supporting the reported results are available at the Kaggle Tabular Playground Series–February 2022 dataset: https://www.kaggle.com/competitions/tabular-playground-series-feb-2022/data (accessed on 10 February 2026).

## Abbreviations

The following abbreviations are used in this manuscript:

BactoRamanBioNet	Bacterial Raman BioNet (model name in the paper)
ResNet1D	Residual Network 1D
CLIP	Contrastive Language-Image Pre-training
SVM	Support Vector Machine
RF	Random Forest
CNN	Convolutional Neural Network
LSTM	Long Short-Term Memory
F1-Score	F1 Score (harmonic mean of precision and recall)
Log-loss	Logarithmic Loss (Logarithmic Loss function)
ReLU	Rectified Linear Unit
BN	Batch Normalization
dk	Dimension of Key (in Transformer)
1D	One-dimensional
L	Sequence Length (feature sequence length)
D	Feature Dimension (dimension of features)

## Appendix A

### Appendix A.1. Model Training and Parameter Initialization

For the model training process, we initialized the parameters of the convolutional and linear layers using the Kaiming initialization method to enhance convergence speed and training stability. The positional encoding within the Transformer module was configured as a learnable parameter, enabling it to adaptively capture the spatial distribution of features as dictated by the task requirements. The entire model was trained in an end-to-end fashion, facilitating the joint optimization of spectral feature extraction, multimodal fusion, and final prediction.

### Appendix A.2. Hardware and Software Environment

The model training and evaluation were conducted on a workstation equipped with an NVIDIA GeForce RTX 3090 GPU with 24 GB of VRAM. The system was powered by an Intel Core i9 series processor. The software environment consisted of Ubuntu 20.58, PyTorch 2.0.0, and CUDA 11.6. We utilized the Hugging Face Transformers library for text feature extraction, alongside standard scientific computing libraries such as NumPy, Pandas, and Torchvision for data processing and analysis. To ensure reproducibility, all experiments were performed under identical hardware and software configurations with fixed random seeds to mitigate the effects of stochasticity.

### Appendix A.3. Ablation Study

To systematically analyze the contribution of the model’s key components, we designed a series of ablation experiments. These experiments investigate three primary aspects: multimodal information integration, feature modeling architecture, and the feature fusion mechanism.

First, to assess the impact of the textual modality, we compared the performance of a unimodal model using only spectral data against our proposed multimodal model that integrates both spectral and text features. Second, to evaluate the role of the self-attention mechanism in capturing global dependencies within spectral features, we compared a baseline model consisting solely of a 1D ResNet against an enhanced model that incorporates a Transformer encoder following the ResNet. Finally, to analyze the effectiveness of different feature fusion strategies, we contrasted a simple concatenation-based fusion followed by a fully connected layer with our proposed approach that employs a Transformer-based fusion module. These experiments are designed to validate the design rationale of each architectural component of the proposed model.
